# A functional amyloid matrix underpins the PDIM-architected corded superstructure of the *Mycobacterium tuberculosis* biofilm

**DOI:** 10.1101/2025.11.07.687260

**Published:** 2026-06-05

**Authors:** Bei Shi Lee, Matthias Godejohann, Richa Mishra, Flavia Gürtler, Abigail J. Deloria, Mengyang Liu, Rainer Leitgeb, Wolfgang Drexler, Vivek V. Thacker, Michael Berney, Richard Haindl

**Affiliations:** 1 Epidemiology, Biostatistics and Prevention Institute, University of Zurich, Zurich, Switzerland; 2 MG Optical Solutions GmbH, Germany; 3 Department of Infectious Diseases, Heidelberg University Medical Faculty, Heidelberg, Germany; 4 Center for Medical Physics and Biomedical Engineering, Medical University of Vienna, Vienna, Austria; 5 Translational Lung Research Centre, Heidelberg University Medical Faculty, Heidelberg, Germany; 6 Albert Einstein College of Medicine, Bronx, USA; 7 Lead contact

**Keywords:** Mycobacterium tuberculosis, Biofilm, Cording, Functional Amyloid, Phthiocerol Dimycocerosate (PDIM), ESX-1 Secretion System, Extracellular Matrix (ECM), QCL-based Mid-Infrared Microspectroscopy, Multimodal Imaging, Phenotypic Drug Tolerance

## Abstract

*Mycobacterium tuberculosis (Mtb)* biofilm formation is associated with antibiotic tolerance, but its architecture remains poorly understood. Here, we reveal that these biofilms form highly-organized superstructures of cords, and through their deconstruction, provide a new molecular insight into *Mtb* biofilms. Using multimodal imaging, we demonstrate that the lipid Phthiocerol Dimycocerosate (PDIM) is required for organizing bacilli into foundational cords and contributes specifically to biofilm-associated antibiotic tolerance. In contrast, the ESX-1 secretion system enhances the biochemical complexity of the extracellular matrix. Notably, we identified a functional amyloid matrix that encases bacterial cords or aggregates within the biofilm, likely conferring structural integrity. Together, these findings support a three-component model that distinguishes structural integrity, physical organization, and biochemical maturation, establishing a new architectural framework for *Mtb* biofilms. Finally, we show that the natural compound epigallocatechin gallate (EGCG) disrupts biofilm formation, highlighting the therapeutic potential of targeting this architecture to overcome drug tolerance in tuberculosis.

## INTRODUCTION

*Mycobacterium tuberculosis* (*Mtb*), the causative agent of tuberculosis (TB), is the leading cause of death from a single infectious agent worldwide^1–4^. Its persistence during treatment reflects not only genetic drug resistance but also phenotypic drug tolerance, in which genetically susceptible bacteria survive antibiotic exposure without acquiring heritable resistance^2,5–7^. This tolerant state is frequently associated with altered metabolic activity and environmental stress, and is increasingly linked to growth within multicellular communities^8–11^. Understanding how these communities are organized is therefore central to overcome therapeutic failure^8,9,12–14^.

A hallmark of *Mtb* multicellular behavior is “cording”, the formation of tight, serpentine bacterial bundles linked to virulence^15–18^. Although the glycolipid trehalose dimycolate (TDM) was historically identified as a “cord factor”^19^, the mechanisms underlying cording are more complex^17^. Both the cell wall lipid phthiocerol dimycocerosate (PDIM)^17,20,21^ and the ESX-1 secretion system^22–26^ have been implicated in this process. PDIM modulates surface hydrophobicity, whereas ESX-1 mediates the secretion of virulence-associated effectors, suggesting coordinated lipid-protein interactions in shaping multicellular organization.

*Mtb* also forms surface-associated biofilms that have been identified in human lung tissue^14^, yet their architecture and molecular organization remain poorly defined. In particular, it is unclear whether and how PDIM and ESX-1 contribute to the formation and structural complexity of these communities.

Here we dissect the architectural and molecular basis of *Mtb* biofilms using genetically modified strains^27–29^ and a multi-modal imaging strategy combining advanced fluorescence microscopy with label-free quantum cascade laser-based mid-infrared microspectroscopy. We find that *Mtb* biofilms form highly organized, corded superstructures encased in a functional amyloid matrix. Within this framework, PDIM is required for the physical organization of bacilli into foundational cords and makes a biofilm-specific contribution to antibiotic tolerance, whereas ESX-1 drives the biochemical complexity of the extracellular matrix.

Together, these findings establish a three-component architectural model, comprising an amyloid matrix that provides structural integrity, PDIM-dependent organization of bacterial assemblies, and ESX-1dependent biochemical maturation. Disruption of amyloid assembly using epigallocatechin gallate impairs biofilm formation, highlighting biofilm architecture as a targetable determinant of drug tolerance in tuberculosis.

## RESULTS

### Avirulent *Mycobacterium tuberculosis* forms submerged biofilms upon exposure to thiol reductive stress

Exposure to thiol reductive stress (TRS) has been shown to be a rapid in vitro trigger for *Mtb* biofilm formation^10^. To assess whether virulence-associated pathways are required for this process, we examined a panel of H37Rv-derived auxotrophic strains (based on mc^²^ 6230 or mc^²^ 6206)^30–33^ differing in their ability to produce PDIM and in the presence or absence of the ESX-1 locus (see Methods).

**Figure 1:**
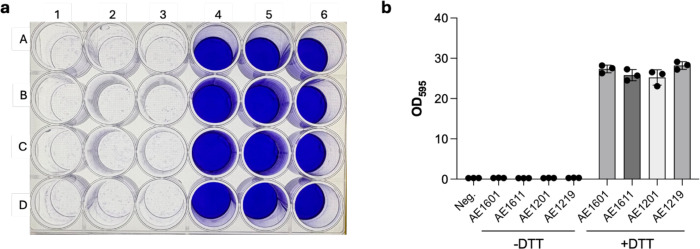
*Mycobacterium tuberculosis* mc^2^ 6230 and mc^2^ 6206 strains form biofilms upon DTT exposure (a) A top view of a 24-well cell culture plate containing (a) AE1601 (PDIM+/ΔRD1); (b) AE1611 (PDIM-/ΔRD1); (c) AE1201 (PDIM+/RD1+); (d) AE1219 (PDIM-/RD1+) following crystal violet (CV) staining. 500 μl of OD-adjusted (to OD_600_ 1.0) culture in detergent-free medium was dispensed in each well. Columns 1–3 contained no DTT while columns 4–6 were supplemented with 6 mM DTT. Following 72 h incubation, the cultures were washed and stained with 0.1% crystal violet. (b) Quantification of biofilm mass by CV destaining. Left to right: negative (no cell) control, AE1601 (-DTT), AE1611 (-DTT), AE1201 (-DTT), AE1219 (-DTT), AE1601 (+DTT), AE1611 (+DTT), AE1201 (+DTT), AE1219 (+DTT). Following the staining step, 95% ethanol was used to solubilize CV. The absorbance at 595 nm was then measured on the spectrophotometer. Values of (+DTT) conditions were calculated based on readings of 20-fold dilutions of the original samples.

Following exposure to 6 mM dithiothreitol (DTT), all strains formed submerged biofilms within 72 hours (Fig. 1a). Despite substantial genetic differences, quantification of total biomass by crystal violet (CV) staining revealed no significant differences across strains (Fig. 1b).

These findings indicate that biofilm initiation under TRS conditions is independent of PDIM and ESX-1 virulence factors, drawing a distinction between early biomass accumulation and structural as well as functional properties defined below.

### PDIM and ESX-1 synergistically dictate a biofilm architecture of a mesh of cords

**Figure 2:**
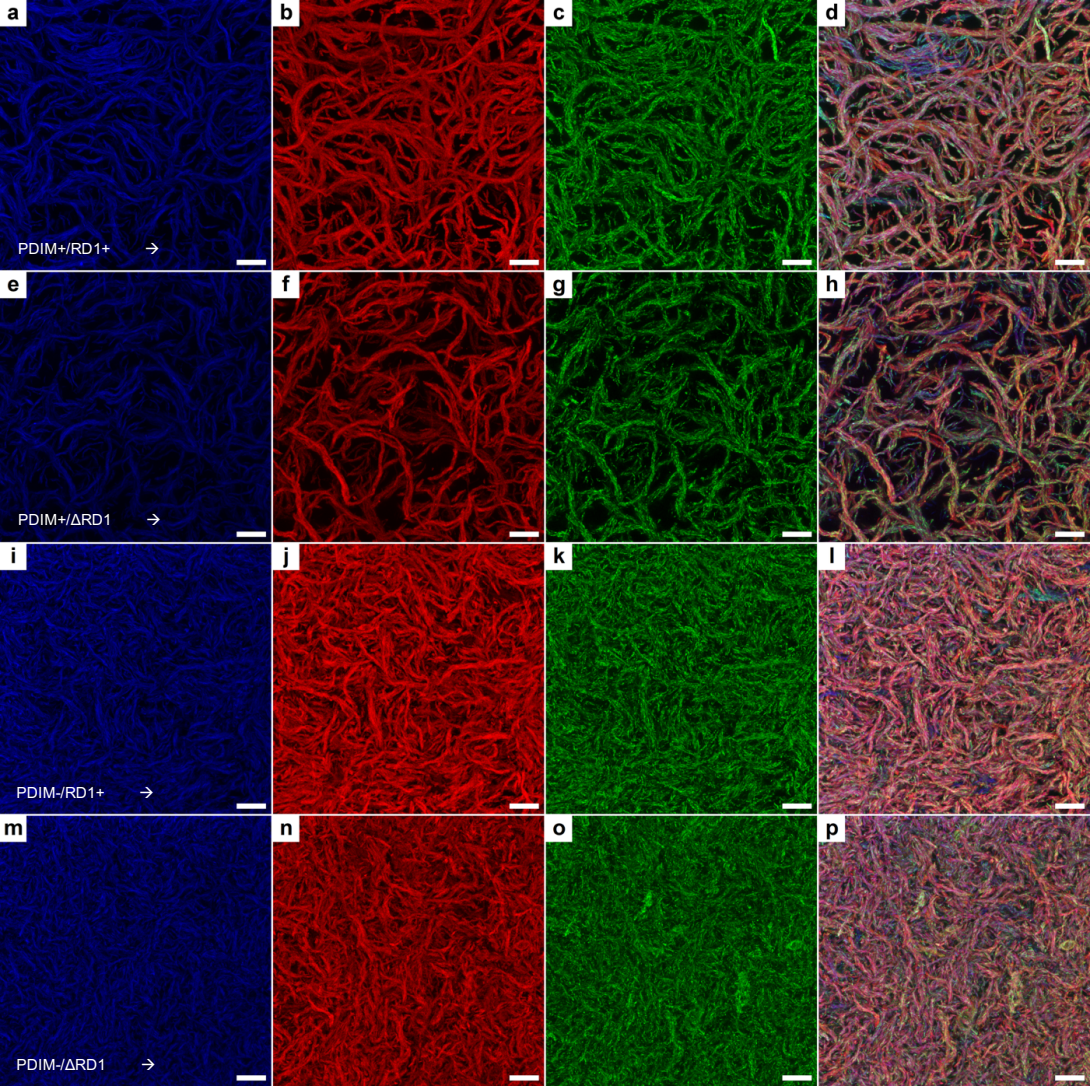
Multi-component fluorescent staining reveals the localization of bacilli within strain-specific biofilm architectures. Confocal microscopy images of paraformaldehyde-fixed biofilms. From left to right, columns display β-polysaccharides (Fluorescent Brightener 28; FB28, blue), α-polysaccharides (Concanavalin A-Alexa Fluor 647; ConA-647, red), nucleic acids (NucGreen Dead 488, green), and a merged image. Rows show representative fields of view for (a-d) AE1201; (e-h); (i-l) AE1219; and (m-p) AE1611. Scale bars, 20 μm.

While the total biomass of the biofilms was comparable across strains (Fig. 1b), their fine-scale organization differed markedly. To resolve these differences, we developed a multi-component staining protocol to simultaneously visualize the key constituents of the biofilm matrix: α- and β-linked polysaccharides, along with the nucleic acids of the resident bacilli (Fig. 2).

This approach revealed that all strains produced matrices containing both polysaccharide classes, which co-localized around the bacteria (Fig. 2d, h, l, p). However, the higher-order organization of these matrix components varied substantially between the strains.

**Figure 3:**
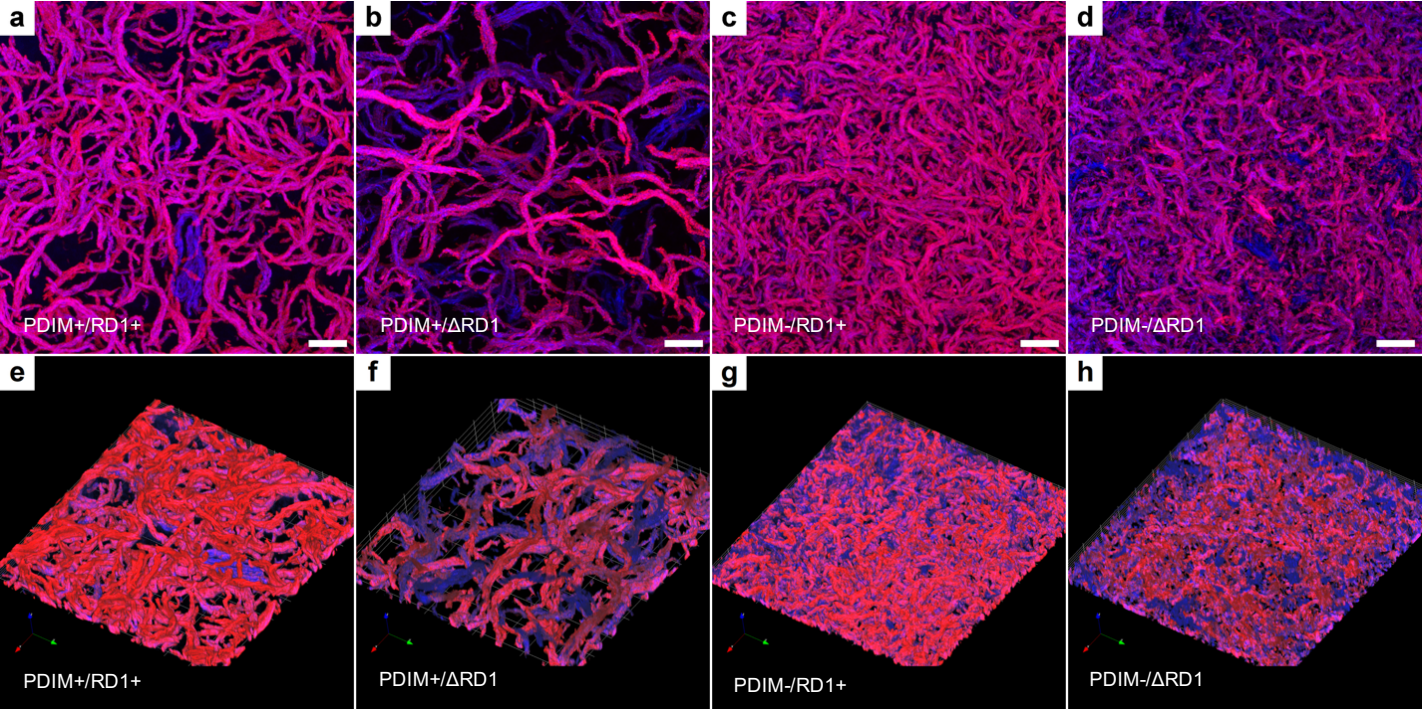
PDIM directs the higher-order assembly of the polysaccharide matrix into a cord-like architecture. A detailed comparative analysis of the polysaccharide scaffold from the biofilms shown in Figure 2. The α-polysaccharide (red) and β-polysaccharide (blue) channels were selected to visualize their combined architecture. The top row (a-d) displays en face maximum intensity projections (MIPs), and the bottom row (e-h) shows corresponding 3D volume renderings. Strains are (a, e) AE1201; (b, f) AE1601; (c, g) AE1219; and (d, h) AE1611. Scale bars, 20 μm.

To examine the polysaccharide scaffold directly, we selected the α- and β-polysaccharide channels and visualized them as both maximum-intensity projections and 3D volume renderings (Fig. 3). In both PDIM-positive strains, AE1201 (PDIM+/RD1+) and AE1601 (PDIM+/ΔRD1), the matrix is made of elongated, well-defined cord-like structures (Fig. 3a, b, e, f). In contrast, PDIM-deficient strains failed to form ordered structures. The AE1219 strain (PDIM-/RD1+) produced a dense, but highly disordered network of shorter, thinner strands (Fig. 3c, g), whereas the double-mutant AE1611 (PDIM-/ΔRD1) formed a compact, “fluffy” matrix of bacterial aggregates (Fig. 3d, h). These data indicate that PDIM is a major determinant of cord-like biofilm macro-architecture.

The comparison between PDIM-positive strains further suggests that ESX-1 contributes to architectural refinement. The wild-type-like AE1201 biofilm appeared denser and more intricate than the AE1601 ΔRD1 biofilm, despite both retaining PDIM (Fig. 3e, f). Thus, while PDIM is required for the emergence of cord-like organization, ESX-1 appears to enhance scaffold consolidation or complexity.

The localization of the bacterial nucleic acids within the image overlay supports this model. In PDIM-positive strains, bacilli were closely aligned and encased within polysaccharide-rich cords (Fig. 2c, g). By contrast, PDIM-negative strains showed more diffuse bacterial organization within poorly defined matrices (Fig. 2k, o). Notably, the AE1611 double-mutant displayed discrete regions of elevated DNA signal (Fig. 2o, p), suggesting that alternative matrix stabilizing features may emerge when both PDIM-dependent organization and ESX-1 activity are absent.

**Figure 4:**
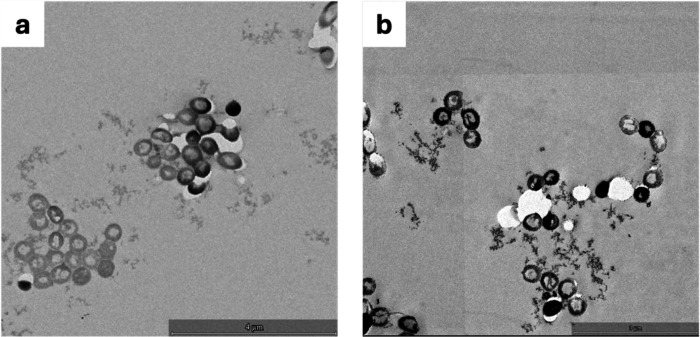
Transmission electron microscopy images of M. tuberculosis biofilms. The images represent sections of biofilms containing *M. tuberculosis* bacilli (measuring approximately 450 nm in diameter). (a) In AE1201, bacilli were closely associated and aligned. Scale bar: 4 μm. (b) In AE1219, the bacilli located were more sparsely associated in a disorganized manner. Scale bar: 3 μm.

The alignment of NucGreen staining along the cord-like structures suggested that these features contain organized bacilli. To directly confirm this, we performed transmission electron microscopy on formaldehyde-fixed biofilms. In PDIM-producing strains, bacilli were arranged in tightly aligned bundles within these structures (Fig. 4a). Cross-sectional views revealed cords composed of at least 5–6 closely packed bacteria across the diameter, with uniform circular profiles consistent with elongated, high-aspect-ratio assemblies. In contrast, bacilli in PDIM-deficient strains lacked this ordered organization and appeared more dispersed with smaller aggregates (Fig. 4b). The morphology and organization of these structures are consistent with the classical description of *Mtb* cording in pellicles and intracellular infections^9,17,18^. Together, these data confirm that the cord-like structures observed in biofilms contain aligned bacilli and represent bona fide cords.

### Mid-infrared imaging reveals an ESX-1-dependent biochemical complexity within the biofilm matrix

**Figure 5:**
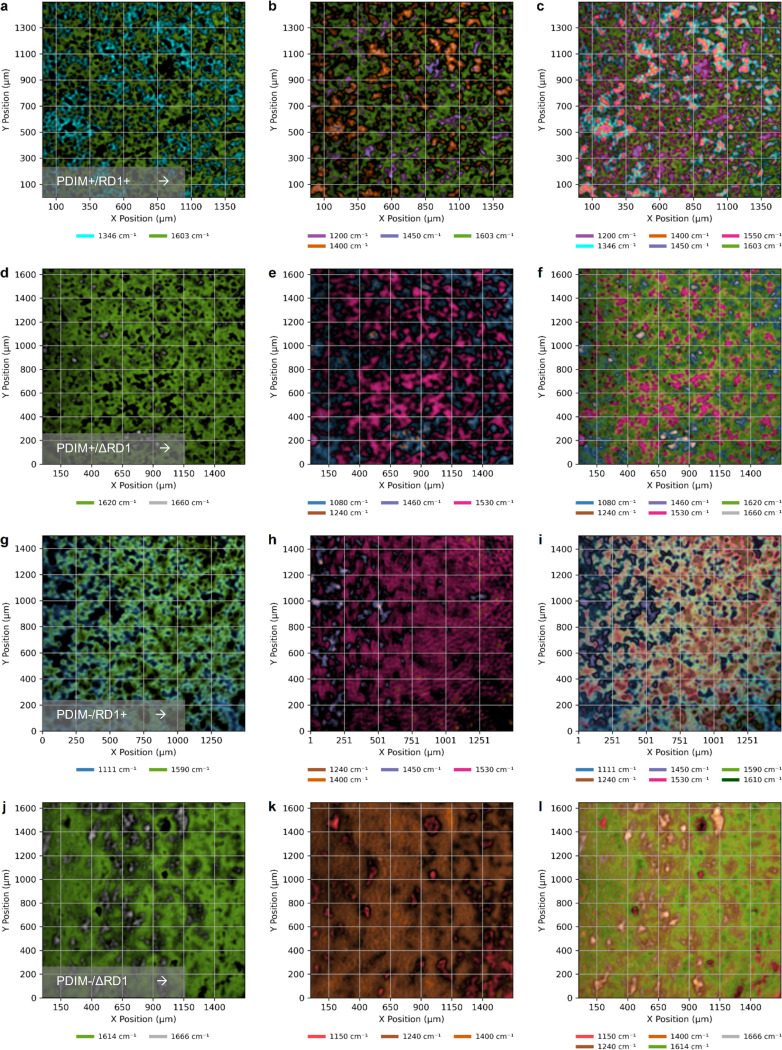
Mid-IR microspectroscopy reveals strain-specific biochemical composition and compartmentalization in *Mtb* biofilms. Chemical maps (Thresholded Specific Wavenumber Abundance Maps, TSWAMs; see Methods) of fixed *Mtb* biofilms imaged in reflection mode. The images show the spatial distribution of specific biomolecular signals, represented by different colors as indicated in the legends within each panel. Each row corresponds to a different strain: (a-c) AE1201; (d-f) AE1601; (g-i) AE1219; and (j-l) AE1611. Columns from left to right show a stepwise, composite visualization of the biochemical architecture. Scale bars are provided within the images.

While fluorescence imaging resolved the physical architecture of the biofilms, it provided little insight into their molecular composition. To map the chemical organization of the matrix, we applied Quantum Cascade Laser (QCL)-based mid-infrared (mid-IR) microspectroscopy to enable label-free visualization of biomolecular components based on inherent vibrational absorption signatures^34,35^. After acquiring hyperspectral data cubes, we generated “Thresholded Specific Wavenumber Abundance Maps” (TSWAMs) to visualize the spatial distribution of key chemical features (Fig. 5; see Methods).

Across all strains, TSWAMs revealed a prominent protein backbone in the Amide I region (1600–1620 cm^−1^, Fig. 5a, d, g, j) indicating a matrix enriched in proteinaceous material. In the wild-type-like AE1201 biofilm, this backbone was primarily associated with a peak at 1603 cm^−¹^, with additional contributions at 1346 cm^−¹^ (Fig. 5a). Incorporation of phosphodiester-associated (1200 cm^−¹^) and lipid-associated (1450 cm^−¹^, 1400 cm^−¹^) signals progressively filled the spatial map, and inclusion of an additional protein-related signal at 1550 cm^−¹^ revealed a highly compartmentalized architecture spanning most of the biofilm (Fig. 5b, c).

This compartmentalization was reduced upon deletion of the ESX-1 locus. In AE1601, the primary protein backbone (~1620 cm^−¹^) remained evident, but regions that were otherwise chemo-spatially distinct in AE1201 were instead largely occupied by polysaccharide (~1080 cm^−¹^) and protein (~1530 cm^−¹^) signals, resulting in diminished fine-scale (Fig. 5d-f).

PDIM-deficient strains exhibited further divergence in chemical organization. The AE1219 biofilm displayed a matrix dominated by Amide II signal at (1530 cm^−¹^), with lipid (~1450 cm^−¹^) and phosphodiester (~1240 cm^−¹^) contributions. Notably, its primary protein signal at 1590 cm^−¹^ overlapped extensively with a carbohydrate-associated peak (1111 cm^−¹^), consistent with a protein-carbohydrate composite scaffold (Fig. 5g-i). The double-mutant AE1611 showed the most extreme phenotype, retaining a protein backbone (~1614 cm^−¹^) but exhibiting a simplified chemical map dominated by a ubiquitous phosphodiester signal at 1240 cm^−¹^, and minimal spatial compartmentalization (Fig. 5j-l).

**Figure 6:**
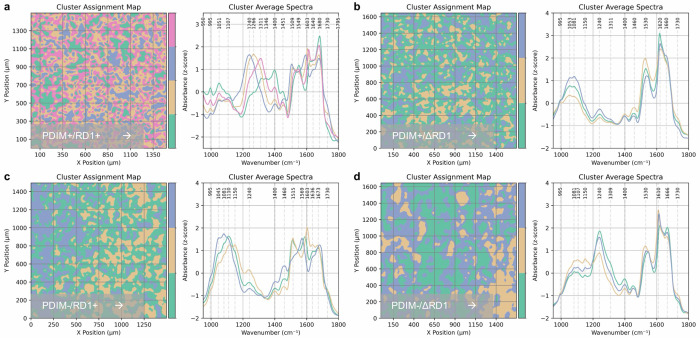
Unbiased clustering defines the biochemical architecture of *Mtb* biofilms. K-means clustering was applied to the mid-IR hyperspectral data to objectively segment the biofilms into distinct chemospatial regions. For each strain, the left panel shows the cluster assignment map, where each color represents a distinct biochemical segment. The right panel shows the corresponding average mid-IR spectrum for each cluster (colored lines). Panels correspond to: (a) AE1201; (b) AE1601; (c) AE1219; and (d) AE1611.

To objectively define these chemo spatial patterns, we applied K-means clustering to the hyperspectral datasets (Fig. 6). The wild-type-like AE1201 biofilm required four distinct clusters to capture its molecular heterogeneity, with cluster spectra rich in diverse protein features and prominent “mixed signal” region (1250–1450 cm^−¹^) (Fig. 6a). In contrast, the ESX-1 deficient AE1601 biofilm was described by three largely redundant clusters, differing mainly in the relative intensity of shared peaks indicating reduced biochemical diversity (Fig. 6b).

Similarly, the PDIM-deficient AE1219 strain exhibited a simplified chemical profile, with diminished contributions from the mixed signal region. Instead, carbohydrate-associated signals are prominent, with z-scores approaching those of the protein bands (Fig. 6c). The AE1611 double-mutant showed minimal spatial complexity but with a striking enrichment of phosphodiester-associated signal (~1240 cm^−¹^) across multiple clusters (Fig. 6d). This phosphodiester signature aligns with the DNA-rich regions observed by fluorescence microscopy (Fig. 2o, p), suggesting that extracellular DNA may assume a more prominent structural role in the absence of both PDIM-dependent organization and ESX-1-driven biochemical maturation. Together, these findings identify ESX-1 as a key driver of biochemical complexity within the *Mtb* biofilm matrix.

### A Proteinaceous Matrix Containing Amyloids Is Essential for Biofilm Integrity

**Figure 7:**
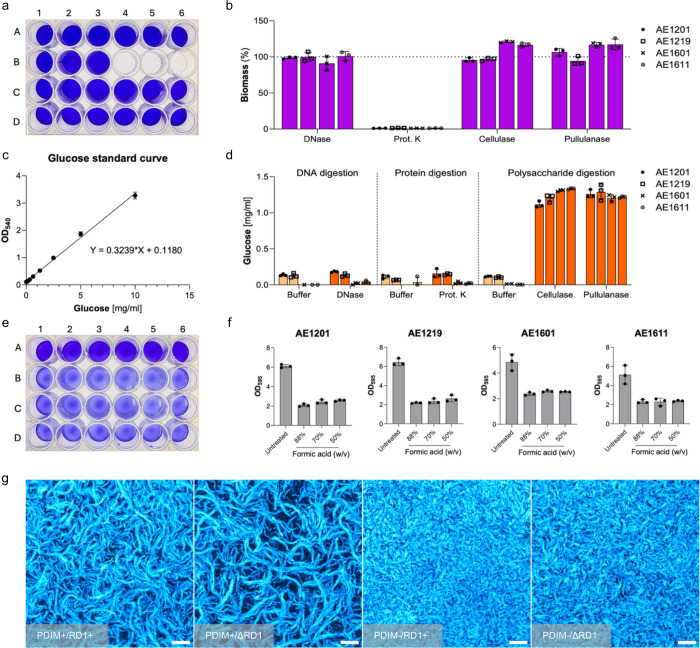
A Functional Amyloid Matrix Is Essential for Biofilm Integrity. (a) Crystal violet-stained biofilms of AE1201. 72 h biofilms of AE1201 were treated with: DNase buffer (wells A1–3), 0.8 U/ml Turbo DNase (A4–6), DPBS (B1–3), 0.1 mg/ml proteinase K (B4–6), 0.1M sodium acetate buffer pH 5 (C1–3), 122 mg/ml cellulase (C4–6), 5 mg/ml cellulase (D1–3), and 4 NPUN/ml pullulanase (D4–6). (b) Quantification of crystal violet staining in *Mtb* biofilms as a reflection of biomass. The biofilms were destained with 95% ethanol and absorbance was measured at 595 nm. Values depicted in the graph are absorbance reads of various enzyme treated biofilms normalised against the mean values from the corresponding buffer only controls. The biofilms were treated with 0.8 U/ml Turbo DNase, 0.1 mg/ml proteinase K, 122 mg/ml cellulase, or 4 NPUN/ml pullulanase. The bars indicate the mean while data points reflect the values from each replicate. (c) A glucose standard curve in a colorimetric assay using 3,5-Dinitrosalicylic acid. The color intensity of the dye was read out as absorbance at 540 nm. (d) Quantification of glucose monomers in the supernatant of enzyme-treated *Mtb* biofilms. (e) Crystal violet-stained biofilms of AE1201 (columns 1–3) and AE1219 (columns 4–6). 72 h biofilms were treated with DPBS (row A), 88% formic acid (row B), 70% formic acid (row C), and 50% formic acid (row D). The biofilms were exposed to the treatment for 1.5–2 minutes before washing and staining with crystal violet. (f) Quantification of crystal violet dye in formic acid treated biofilms. (g) Confocal microscopy images of the four *Mtb* strains stained with Thioflavin T (ThT) to visualize functional amyloids. A strong fluorescent signal is present in all strains, appearing as an organized, cord-like network in PDIM-proficient strains (AE1201, AE1601) and as a more disorganized mesh in PDIM-deficient strains (AE1219, AE1611). Scale bars, 20 μm.

To identify the key structural components of the biofilm, we subjected biofilms to enzymatic digestions (Fig. 7a, b). Proteinase K treatment caused complete detachment of the biofilms across all strains, demonstrating that the extracellular matrix critically depends on a proteinaceous scaffold, consistent with prominent protein signatures observed by mid-IR microspectroscopy (Fig. 5, 6). Examination of the biofilm fragments from the digestion under microscopy revealed that cord structures were retained, showing that proteins are involved in biofilm attachment rather than cord formation (data not shown). In contrast, treatment with DNase, pullulanase (commercial type I/type II preparation targeting α−1,6 and α−1,4 glucosidic linkages), and cellulase did not disrupt overall biofilm structure (Fig. 7a, b). Nevertheless, biochemical analysis of digestion supernatants confirmed the presence of polysaccharide substrates, as pullulanase and cellulase treatments released significant quantities of sugar monomers (Fig. 7c, d).

Given the essential role of the protein matrix and the prominent anti-parallel β-sheet-rich spectral signatures detected by mid-IR analysis (Fig. 6)^36^, we next investigated whether amyloid structures contribute to biofilm stability. Treatment with formic acid, which dissolve amyloid aggregates^37^, caused a significant, dose-dependent reduction in biofilm biomass (Fig. 7e, f). To directly visualize these structures, biofilms were stained with the amyloid-specific dye Thioflavin T (ThT)^38^. Confocal microscopy imaging revealed strong ThT signal co-localizing with bacterial cords or aggregates across all strains (Fig. 7g), indicating that amyloid matrix formation occurs independently of PDIM production and ESX-1 function. Together, these findings identify amyloids as a central structural component required for maintaining *Mtb* biofilm integrity.

**Figure 8:**
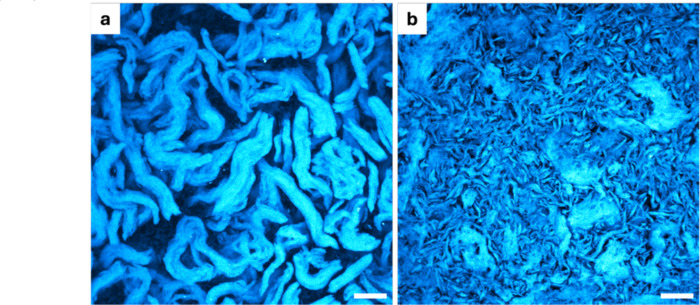
Thioflavin T staining of *M. tuberculosis* Erdman biofilms. (a) Wildtype *M. tuberculosis* Erdman forms a biofilm when exposed to DTT-induced thiol reductive stress. Thick, serpentine cords are observed in the biofilm architecture, which also stained positively for amyloid proteins. (b) A *M. tuberculosis* Erdman *Tn:fadD26* mutant which is incapable of PDIM biosynthesis, showed a biofilm matrix with short, disorganized aggregates. Scale bars, 20 µm.

To determine whether these architectural features extend beyond avirulent laboratory strains, we next examined fully virulent *M. tuberculosis* Erdman under the same reductive stress conditions. Erdman formed a submerged biofilm containing thick, serpentine cords that stained strongly with ThT, revealing a corded amyloid positive matrix comparable to that observed in the avirulent strains (Fig. 8a). We further examined an Erdman mutant carrying a transposon insertion in *fadD26* which abolishes PDIM biosynthesis^39,40^. In contrast to wild-type Erdman, the *Tn:fadD26* mutant failed to form robust cord structures and instead produced disordered bacterial aggregates (Fig. 8b). Thus, both the PDIM-dependent cord architecture and the amyloid-associated matrix are conserved between avirulent and virulent *Mtb* strains. These findings further support the use of BSL-2 compatible strains as safer surrogate models for studying biofilm architecture and cording.

### Biofilm superstructures enhance antibiotic tolerance

**Extended Data Figure 1:**
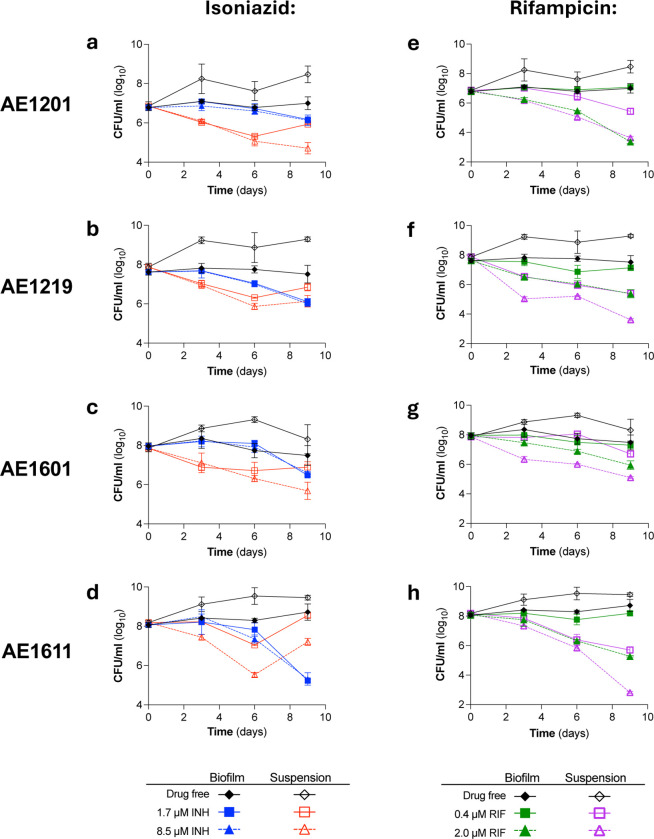
The formation of biofilm protects *Mtb* from antibiotic treatment across all four genetic backgrounds. *Mtb* cultures in either 7H9 broth or in 3-day-old submerged biofilms were treated with isoniazid or rifampicin, and bacterial viability was quantified at the start of the experiment (day 0), day 3, day 6, and day 9. AE1201 (a), AE1219 (b), AE1601 (c), and AE1611 (d) were treated with 1.7 µM (square symbols with solid line) or 8.5 µM isoniazid (triangle symbols with dash line). AE1201 (e), AE1219 (f), AE1601 (g), and AE1611 (h) were treated with 0.4 µM (square symbols with solid line) or 2.0 µM rifampicin (triangle symbols with dash line). In all graphs, drug free controls are depicted as black diamonds with solid line.

**Figure 9:**
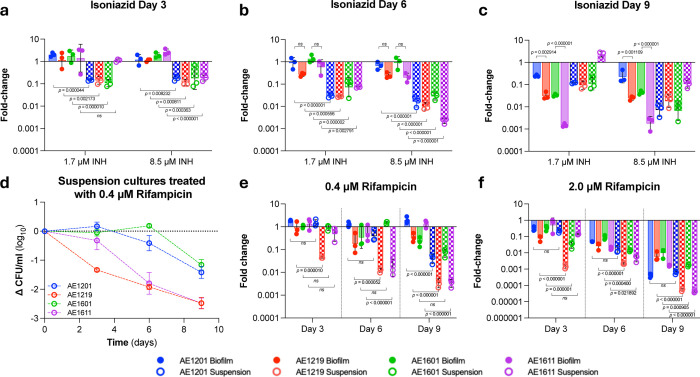
Differential antibiotic tolerance of *M. tuberculosis* strains in planktonic and biofilm states. Four *M. tuberculosis* strains (AE1201, AE1219, AE1601, AE1611) were exposed to isoniazid (INH) or rifampicin (RIF) at two concentrations each, and viable bacteria were quantified by CFU enumeration on days 3, 6, and 9. a-c: INH treatment at 1.7 µM and 8.5 µM, shown for day 3 (a), day 6 (b), and day 9 (c). d: Kinetics of planktonic (suspension) cultures exposed to 0.4 µM RIF, plotted as the change in CFU/ml over time. e-f: RIF treatment at 0.4 µM (e) and 2.0 µM (f) across day 3, day 6, and day 9. For panels (a–c, e–f), CFU are plotted as fold-change relative to the corresponding untreated control for each strain, time point, and growth state. Filled symbols denote biofilm-derived CFU and open symbols denote suspension-derived CFU (color-coded by strain). Bars indicate group means; points represent individual replicates, and error bars denote standard deviation. Statistical significance was assessed using one-way ANOVA with Tukey’s multiple-comparisons test; adjusted *p* values are shown on the plots, and “*ns*” indicates no significant difference.

To determine whether differences in the biofilm architecture influence antibiotic protection, biofilms were exposed to high concentrations of frontline antibiotics isoniazid (INH) and rifampicin (RIF) and viability was monitored over nine days (Fig. 9, Extended Data Fig. 1). In parallel, the same strains were treated under planktonic growth conditions in the absence of DTT and presence of tyloxapol (Extended Data Fig. 1, red symbols and lines) to distinguish biofilm-specific effects from intrinsic drug susceptibility.

**Extended Data Figure 2:**
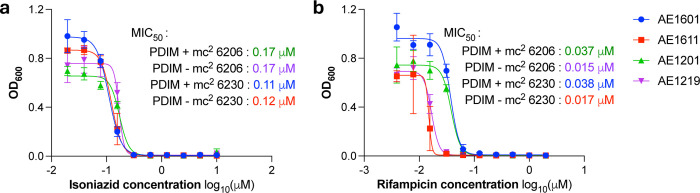
Minimum inhibitory concentration of isoniazid and rifampicin in avirulent *Mtb* strains. *M. tuberculosis* strains AE1601 (blue circles), AE1611 (red squares), AE1201 (green triangles), and AE1219 (purple triangles) were incubated with various concentrations of isoniazid (a) or rifampicin (b) for 10 days and growth was recorded as optical density at 600 nm. MIC_50_: minimum antibiotic concentration to achieve 50% growth inhibition.

INH was tested at 1.7 µM and 8.5 µM, corresponding to approximately 10x and 50x the MIC_50_ values of the strains (Extended Data Fig. 2a). As expected, PDIM and RD1 status did not alter intrinsic susceptibility to INH. Under planktonic conditions, all strains exhibited the characteristic rapid decline in viability followed by outgrowth after prolonged exposure consistent with the emergence of escape mutants (Extended Data Fig. 1a-d)^41^. In contrast, biofilm-associated bacteria showed markedly delayed susceptibility to INH (Fig. 9a and b, Extended Data Fig. 1a-d). This protective effect was strongly influenced by PDIM expression: biofilms formed by PDIM positive strains retained much greater protection against INH (Fig. 9b and c).

Biofilm-associated protection was also observed during RIF treatment (Fig. 9d, Extended Data Figure 1e-h). However, interpretation of these effects was complicated by the known inherent differences in RIF susceptibility associated with PDIM status^42^ (Extended Data Fig. 2b) At standardized concentrations of 0.4 µM and 2.0 µM RIF, PDIM-positive planktonic cultures already displayed increased survival relative to PDIM-deficient strains, reducing the apparent contribution of the biofilm state itself. Nevertheless, biofilm-associated protection became more evident at later time points and was most apparent in the PDIM-deficient strains AE1219 and AE1611 (Fig. 9d), where intrinsic resistance contribution was lower.

Together, these findings demonstrate that *Mtb* biofilms confer substantial antibiotic tolerance and further identify PDIM-dependent biofilm architecture as an important contributor to protection against antibiotic stress.

### EGCG impairs *Mtb* biofilm formation

**Extended Data Figure 3:**
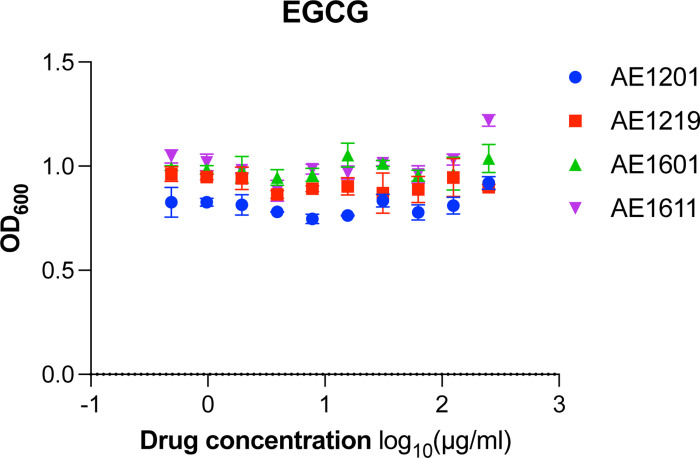
Growth of *Mtb* strains in liquid culture were unaffected by Epigallocatechin gallate (EGCG) exposure. AE1201 (blue circles), AE1219 (red squares), AE1601 (green triangles), and AE1611 (purple triangles) were exposed to various concentrations of EGCG for 10 days before culture density was measured in a plate spectrophotometer (absorbance at 600 nm). Data points reflect average value of triplicate measurements, and error bars indicate standard deviation.

**Figure 10:**
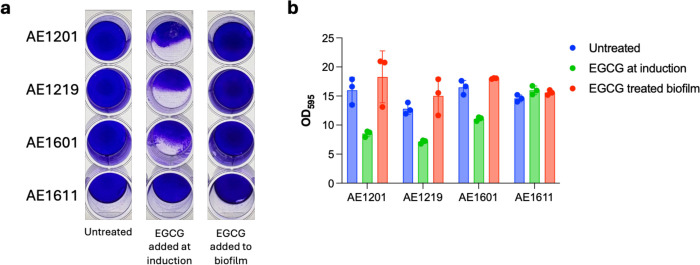
The effect of epigallocatechin gallate (EGCG) on biofilm formation of *Mtb*. (a) Left to right: untreated, 250 µg/ml EGCG added at induction of biofilm, 250 µg/ml EGCG added to 72h mature biofilm. The Mtb strains are (from top to bottom row) AE1201, AE1219, AE1601, and AE1611. The biofilms were stained with 0.1% crystal violet 4 days after the formed biofilms were exposed to EGCG. (b) Quantification of crystal violet staining in EGCG treated *Mtb* biofilms as a reflection of biomass. The columns are grouped by strain, and untreated biofilm is represented in blue, biofilm treated with EGCG at induction is in green, and mature biofilm treated with EGCG is represented in red.

Given the evidence supporting a structural role for amyloids within the *Mtb* biofilm matrix, we next examined whether disruption of amyloid assembly affects biofilm formation. To test this, we used (−)-epigallocatechin gallate (EGCG), a polyphenol compound with established anti-amyloidogenic activity that inhibits the formation of b-sheet rich amyloid fibrils and destabilize preformed aggregates^43^. Importantly, EGCG exhibited no detectable inhibitory effect on planktonic *Mtb* growth in vitro at concentrations up to 250 µg/ml (Extended Data Fig. 3), allowing assessment of biofilm-specific effects independent of direct antibacterial activity. EGCG was therefore added either at the time of biofilm induction together with DTT, or after biofilm maturation following 72h of reductive stress exposure. Biofilm biomass was quantified four days later with crystal violet staining (Fig. 10a, b). Addition of EGCG during biofilm induction significantly impaired biofilm formation in all strains except the PDIM/ESX-1 double mutant AE1611(Fig. 10a, b). In contrast, treatment of pre-established biofilms produced no detectable reduction in crystal violet staining over the experimental time course.

These findings indicate that the amyloid component contributes to early biofilm establishment and further support a central architectural role for amyloid-containing matrices in *Mtb* biofilm formation.

## DISCUSSION

The ability of *Mycobacterium tuberculosis* to form resilient biofilms represents a major obstacle in tuberculosis treatment, yet the molecular principles governing their complex architecture remain poorly defined. Here, we show that genetically distinct strains lacking major virulence determinants retain the ability to form biofilms but exhibit profound differences in their physical and biochemical organization. Using a multi-modal imaging approach, we define a division of labor between three systems that together generate the mature *Mtb* biofilm architecture: PDIM directs the physical, cording-like macrostructure; a functional amyloid matrix provides the structural integrity; and the ESX-1 secretion system biochemical diversity of the extracellular matrix. The mature wild-type-like biofilm therefore emerges from the coordinated interplay of these distinct architectural layers.

### PDIM-directed cording underlies *Mtb* biofilm architecture

Our imaging analysis reveals that submerged *Mtb* biofilms are composed of highly ordered multicellular structures in which bacilli reside. PDIM expression was the dominant determinant of this phenotype: PDIM-positive strains formed long, thick cord-like structures, whereas PDIM-deficient strain produced dense but disorganized short filaments or aggregates. The morphology, dimensions, and bacterial organization of these structures are consistent with classical descriptions of *Mtb* cording observed in pellicles and infected macrophages^18,44^. We therefore ascertain that the rope-like structures within the biofilm are, in fact, organized cords. Historically, cording has been attributed to trehalose dimycolate (TDM), the canonical “cord factor”^19^. However, our findings indicate TDM alone is insufficient to generate higher-order cord architecture. PDIM-deficient strains retained TDM production yet failed to organize into elongated cords, demonstrating that additional membrane-associated determinants are required for stable multicellular alignment. PDIM is a major lipid component of the mycomembrane and is known to alter membrane biophysical properties^22,45^. It is thus possible that both PDIM and TDM is necessary to maintain a membrane environment that supports cording.

### An amyloid matrix provides structural integrity to the biofilm

Although PDIM dictated the overall architecture of the biofilms, proteinaceous components are essential for maintaining its structural cohesion. Proteinase K treatment caused complete detachment and collapse of biofilms across all strains, whereas DNase and polysaccharide-degrading enzymes digestions did not disrupt matrix integrity despite biochemical evidence of abundant polysaccharides within the extracellular matrix. These findings indicate that proteins constitute the primary load-bearing scaffold of the biofilm.

Mid-IR analysis further revealed prominent anti-parallel β-sheet-associated signatures (~1605–1630 cm^−¹^ and ~1680 cm^−¹^)^−¹^)^46–50^, together with weaker parallel β-sheet contributions (~1640 cm^−¹^)^50^ particularly in the RD-1 intact strains. Such extracellular β-sheet enrichment is characteristic of cross-β amyloid assemblies^51–53^. Consistent with this interpretation, Thioflavin T (ThT) strongly stained the extracellular cord structures. ThT exhibits a massive increase in quantum yield only when its rotation is immobilized within the rigid channels of an amyloid cross-β structure^54^. While ThT can bind to eDNA, such interactions result in only a weak fluorescence enhancement and do not account for the intense signal observed^55^. Furthermore, formic acid rapidly disrupts the extensive hydrogen-bonding network that stabilizes the cross-β core of amyloid fibrils^56^. Its ability to disaggregate the matrix in the matter of 1–2 minutes is consistent with the physical disaggregation of amyloid fibrils via disruption of non-covalent bonds^56^, a process orders of magnitude faster than the covalent modification of general proteins or acid-catalyzed hydrolysis of polysaccharides^57^.

Together, these orthogonal observations identify amyloid-like polymers as a major structural component of the extracellular matrix. The convergence of spectroscopic, chemical, enzymatic, and imaging data therefore strongly supports the presence of a functional amyloid scaffold within the *Mtb* biofilm matrix.

Importantly, amyloid formation occurred independently of both PDIM and ESX-1, indicating that amyloid deposition represents a foundational and genetically separable layer of biofilm organization. We therefore propose a model in which a protease-resistant amyloid scaffold provides inter-cord cohesion, while protease-sensitive protein interactions anchor this framework to the surrounding surfaces.

While the molecular identity of this amyloid remains to be definitively established, the *Mtb* protein MTP (Rv3312A), which forms curli-like pili, is a possible candidate^58^. Other stress-response proteins with βsheet potential, like HspX^1,56,57^ or GroEL^9,11,59^, could also contribute to this framework. For instance, GroEL2, beyond its canonical function, is known to be surface-exposed and acts as an adhesin that mediates binding to host cells by interacting with lectin-like receptors^60^.

### ESX-1 *drives biochemical maturation of the extracellular matrix*

While ESX-1 is dispensable for amyloid formation and cord assembly, it strongly influenced the biochemical complexity of the biofilm matrix. Mid-IR mapping and K-means clustering demonstrated that RD1-intact strains exhibited substantially greater chemospatial heterogeneity and compartmentalization than their DRD1 counterparts, suggesting that ESX-1 contributes primarily to matrix maturation. Furthermore, transcriptomic data conducted by Trivedi et al. highlighted that ESX-1 was found to be upregulated in biofilm-dwelling *Mtb*, further corroborating the involvement of this secretion system in *Mtb* biofilm formation^10^. This increased complexity may arise from the secretion of ESX-1 substrates like EspB, which is known to self-assemble into stable oligomers that could integrate into and diversify the primary amyloid network^61^.

### Polysaccharides form an integrated scaffold

A notable feature of the biofilm matrix is the close spatial association between polysaccharides and the bacterial cords. Rather than forming a diffuse layer, a- and b-linked polysaccharides enveloped the cord structures and co-localized with the extracellular scaffold. The prominent α-polysaccharide signal, stained by Concanavalin A, is consistent with the abundance of glycogen-like α-glucans in the mycobacterial capsule^62,63^.

These polysaccharides likely contribute more than passive encapsulation. Analogous to other microbial systems^64^, extracellular glycans may stabilize or spatially organize the amyloid matrix around bacterial cord assemblies. While mycobacterial lectins are less characterized, genomic analysis predicts proteins with homology to these fungal adhesins^65^, and specific mycobacterial proteins are known to bind endogenous cell wall glycolipids^66^. The resulting matrix therefore appears to function as an integrated composite material in which polysaccharides, amyloid-like fibrils, and lipid-directed cord structures are physically interwoven. Together, these findings define the *Mtb* biofilm as a highly engineered composite material with distinct but cooperative architectural components.

### Biofilm architecture contributes to antibiotic tolerance

Direct comparison of planktonic and biofilm-associated bacteria demonstrated that the extracellular matrix confers substantial protection against antibiotic exposure. For isoniazid, intrinsic susceptibility was unaffected by PDIM or ESX-1^42^, allowing biofilm-specific effects to be distinguished from cell-intrinsic resistance mechanisms. Under these condition, PDIM-positive biofilms were more tolerant to antibiotics than PDIM-deficient biofilms, indicating that PDIM-directed architecture itself contributes to antibiotic tolerance. These findings directly link higher-order biofilm organization with the protective capacity of the extracellular matrix.

A likely explanation is that the extracellular matrix acts as a diffusion barrier that limits antibiotic penetration^67,68^. Although altered physiology may also contribute^69–71^, transcriptomic profiling indicated that biofilm-dwelling *Mtb* remain metabolically active^10^, suggesting that the physical barrier of the biofilm matrix is the dominant factor of antibiotic tolerance. In addition to the outer mycomembrane, PDIM has also been reported in mycobacterial extracellular vesicles (MEV)^72^. Given that extracellular vesicles influence biofilm formation in other bacteria including *Mycobacterium ulcerans*^73,74^, it is plausible that PDIM is present within the extracellular matrix of *Mtb* biofilm and contributes to modulating the protective properties of the matrix against small-molecule drugs.

### EGCG disrupts *Mtb* biofilm formation

In Alzheimer’s disease, EGCG prevents the accumulation of β-sheet rich amyloid fibrils by 1. binding to unfolded polypeptides and preventing aggregate formation, and 2. directly binding β-sheet rich fibrils and remodeling into amorphous aggregates^75,76^. Our observation that EGCG impaired *Mtb* biofilm formation reinforces our finding that functional amyloids are involved in the *Mtb* biofilm matrix. Beyond *M. tuberculosis*, EGCG has also been reported to inhibit biofilm formation in other bacteria, including *Shigella flexneri* and *Enterococcus faecalis*^77,78^. In these cases, however, the compound is thought to act, at least in some contexts, by triggering oxidative stress (e.g., hydroxyl radical production and H_₂_O_₂_ generation) intracellularly at high concentrations. While such responses could, in principle, chemically counteract the reductive effects of DTT and thereby inhibit biofilm formation, the EGCG concentration used here is insufficient to measurably inhibit *M. tuberculosis* growth; thus, it is unlikely this is the mechanism in which EGCG acts against *Mtb*. Additionally, while EGCG may partially counteract DTT through redox interactions, complete quenching of the DTT biofilm-inducing effect appears unlikely. DTT was present at an approximately 11-fold molar excess over EGCG, and the concentrations remaining even after partial neutralization would still be expected to fall within the range previously reported to induce mycobacterial biofilm formation^10^. This suggests that the inhibitory effect of EGCG is not due to depletion of DTT but likely involves a more direct interference with biofilm formation. The mechanism underlying AE1611’s resistance to EGCG remains unclear. Nevertheless, mid-IR imaging and confocal NucGreen staining point to a more prominent extracellular DNA component in the AE1611 biofilm. This may indicate that differences in matrix composition reduce the dependence of AE1611 on amyloids during biofilm formation. Interestingly, EGCG has been reported to exert host-directed effects that can support clearance of *M. tuberculosis* infection^79^, suggesting an additional dimension to its activity that may complement antibiotic treatment and merit further investigation.

### Implications and Future Perspectives

Our data collectively argue that the *Mtb* biofilm architecture is arising from the synergy between three distinct systems. We show that the lipid PDIM directs the physical, corded scaffold; a fundamental functional amyloid matrix provides the overall structural integrity; and the ESX-1 secretion system drives the final biochemical complexity of this matrix. A key advance enabling this framework was the application of the QCL-based mid-IR microspectroscopy to *Mtb* biofilms, which provided a label-free view of their native chemical landscape. Future studies should identify the proteins that constitute the functional amyloid matrix and define the ESX-1-dependent extracellular components that contribute to biochemical complexity.

Our findings also reveal novel therapeutic vulnerabilities. Rather than targeting bacterial viability directly, disrupting extracellular architecture may sensitize biofilm-associated *Mtb* to conventional antibiotics. The ability of EGCG to impair biofilm formation highlights the feasibility of targeting amyloid assembly as an anti-biofilm strategy. Finally, the discovery that submerged *Mtb* biofilms form highly organized corded superstructures establishes this system as a tractable model for studying mycobacterial cording and multicellular assembly. Future work should also aim to develop a multi-scale biophysical model to test how the interplay between PDIM-driven hydrophobic aggregation and specific TDM-mediated interactions drives the assembly of mycobacterial cords independent of cell growth^18^.

## Conclusion

In summary, our work deconstructs the architecture of the *Mtb* biofilm and reveals that these communities are not amorphous aggregates but highly organized composite structures. We demonstrate that PDIM directs cord formation, amyloids provide structural integrity, and ESX-1 drives biochemical maturation of the extracellular matrix. This insight shifts the focus of therapeutic intervention from targeting the bacterium alone toward dismantling the protective biofilm architecture.

### Limitations of the study

This study has several limitations that frame the context of our findings. Experiments were performed in a static, *in vitro* culture system. Biofilm architecture within infected hosts is likely influenced by immune pressure, nutrient limitation and dynamic microenvironmental conditions^10,14,80–82^. Future studies will therefore be required to determine whether these architectural principles are conserved across clinical isolates and in vivo infection models.

Second, although QCL-based mid-IR microspectroscopy provides powerful chemospatial information on proteins, lipids, polysaccharides, and phosphodiesters^83,84^, it does not identify specific molecular species^85,86^. Definitive characterization of the amyloid matrix and ESX-1-associated extracellular components will require complementary techniques such as spatially-resolved proteomics^86–88^.

### RESOURCE AVAILABILITY

#### Lead contact

Further information and requests for resources and reagents should be directed to and will be fulfilled by the lead contact, Richard Haindl (richard.haindl@meduniwien.ac.at).

## METHODS

### EXPERIMENTAL MODEL AND STUDY PARTICIPANT DETAILS

#### Bacterial Strains

All avirulent *Mycobacterium tuberculosis* strains used in this study are derived from H37Rv background. AE1601 and AE1611 are pure isolated PDIM-positive and PDIM-negative clones, respectively, selected from H37Rv mc^2^ 6230 (ΔRD1 Δ*panCD*)^42^. AE1611 has an additional *ppsC* c.2685(C)7→8 frameshift mutation that results in PDIM deficiency. AE1201 and AE1219 are pure isolated PDIM-positive and PDIM-negative clones, respectively, selected from H37Rv mc^2^ 6206 (Δ*leuCD* Δ*panCD*). AE1219 has a point mutation in *fadD26* (Trp43*) that results in PDIM deficiency.

Virulent BSL-3 *Mtb* strains (WT and *Mtb* 5’Tn::*fadD26)* were derived from *Mtb* Erdman^89^.

#### Culture Conditions

All avirulent strains were routinely cultured using Middlebrook 7H9 base (0.2% glycerol, 0.05% tyloxapol, 10% OADC (oleic acid, bovine serum albumin, dextrose, catalase, sodium chloride)). The cultures were supplemented with 24 µg/ml calcium pantothenate and 50 µg/ml L-leucine as needed; PDIM-positive cultures are further supplemented with 0.1 mM sodium propionate.

Culturing of all BSL-3 mycobacterial strains was performed at 37°C in Middlebrook 7H9 base (Difco) supplemented with 0.5% albumin, 0.2% glucose, 0.085% sodium chloride, 0.5% glycerol, 0.02% tyloxapol in 30 mL inkwell bottles with shaking at 200 rpm.

#### Biofilm Induction

To induce biofilm formation in vitro, logarithmic phase bacterial cultures were centrifuged at 3500 ×g for 10 minutes at room temperature. The cell pellet was washed once with biofilm medium (Middlebrook 7H9 base, 0.2% glycerol, 5% OADC) and cell density was adjusted to OD_600_ 1.0 in the same medium. Supplements (calcium pantothenate, L-leucine, and sodium propionate) were added as necessary. 6 mM dithiothreitol (DTT) was added to the culture and mixed thoroughly before dispensing into various forms. Samples for fluorescence confocal microscopy were cultured in ibidi µ-Slide 8 Well high plates. For mid-IR microscopy, autoclaved Kevley slides were aseptically placed in sterile 100 mm × 15mm petri dishes. 300 µl of culture was dispensed per well in the ibidi slides, and 25 mL in each petri dish. The plates or slides were incubated at 37 °C in static, humidified conditions.

#### Biofilm Fixation

Freshly prepared 4% paraformaldehyde (PFA) was used to fix *Mycobacterium tuberculosis* biofilms. The culture media was first carefully removed, then washed twice with DPBS (Corning, 21–031-CV). After DPBS was removed, 4% PFA was dispensed to completely submerge the biofilm and incubated for one hour at room temperature. Following fixation, the fixing solution was removed, and samples were washed twice with sterile water and stored in DPBS at 4°C, protected from light, until staining. Samples for mid-IR imaging were air dried overnight before storing at room temperature, away from light, until ready for measurement. The fixation protocol was validated prior to the removal of samples from the BSL-2 certified laboratory for downstream imaging steps. PFA-treated biofilms were washed twice with sterile water and then scraped off from the wells using wide-bore pipette tips. The entire content was then plated on agar plates (Middlebrook 7H10, 0.5% glycerol, 10% OADC, 24 µg/ml calcium pantothenate and 50 µg/ml L-leucine, 1 mM sodium propionate). The agar plates were incubated at 37 °C for 4 weeks to confirm that no viable bacteria were present.

### METHOD DETAILS

#### Quantification of Biofilm Biomass by Crystal Violet Staining

Following a 72h induction period, supernatant in each well containing *Mtb* biofilm was carefully removed by pipetting. The biofilms were washed twice with sterile DPBS before adding 500 µl of 0.1% crystal violet solution into each well. The wells were incubated with the stain for 30 minutes at room temperature. After 30 minutes, the staining solution was removed and washed thrice with DPBS to remove excess dye. To extract biofilm-bound crystal violet, 500 µl 95% ethanol was added to destain for 30 minutes at room temperature. 200 µl of the ethanol solution was added to a 96-well flat bottom microtiter plate (or diluted as needed) and absorbance at 595 nm was measured using a spectrophotometer.

#### Enzymatic Treatment of Biofilms

Following a 72h induction period, supernatant in each well containing *Mtb* biofilm was carefully removed by pipetting. The biofilms were washed twice with sterile DPBS. For DNase treatment, the buffer was prepared by diluting the 10X buffer provided in the Turbo DNase kit with sterile water. Proteinase K was added to DPBS (pH 7.4). Cellulase and pullulanase were diluted in 0.1 M sodium acetate buffer at pH 5. The enzymes were adjusted to the stated concentrations in their respective buffers, and 500 µl aliquoted into each well containing the washed biofilms. The plates were then incubated for 24 hours at 37 °C. After 24 hours, the supernatant was sampled for the quantification of glucose. The remaining supernatant was removed, and the biofilms were washed twice with sterile DPBS before crystal violet staining.

#### DNS (3,5 –dinitrosalicylic acid) Colorimetric Assay for Reducing Sugar Quantification

The DNS solution was prepared as such: 1.0 g of DNS was added to 50 ml of distilled water and heated to 90°C until dissolved. The solution was allowed to cool, and 20 ml of 0.1 g/ml NaOH was added. 30 g sodium potassium tartrate was added gradually to the solution. Finally, the volume was adjusted to 100 ml using distilled water.

50 µl of a sample (biofilm supernatant or glucose standards) was added to equal volume of the DNS solution and heated at 95°C for 10 minutes. 50 µl of the cooled samples were then added to 50 µl of water, and the absorbance at 540 nm was then measured using a spectrophotometer.

#### Preparation of Staining Reagents

##### Concanavalin A, Alexa Fluor^™^ 647 (ConA-647) Stock Solution (2 mg/mL):

2 mg of Concanavalin A, Alexa Fluor^™^ 647 Conjugate was dissolved in 1 mL of 0.1 M sodium bicarbonate buffer (pH 8.5). The ConA-647 solution was mixed by gentle vortexing and then centrifuged at 300 ×g for 1 minute. The supernatant was aliquoted and stored at −20°C, protected from light.

##### ConA-647 Working Solution (200 µg/mL):

A frozen aliquot of the 2 mg/mL ConA-647 stock solution was thawed and vortexed gently. 100 µL of the stock solution was diluted in 900 µL of DPBS containing calcium and magnesium. The working solution was mixed by gentle vortexing and used fresh.

##### Fluorescent Brightener 28 (FB28) Stock Solution (250 mg/mL):

Fluorescent Brightener 28 Disodium Salt Solution, supplied as a 25% (w/v) solution in water, was used as the stock solution.

##### FB28 Working Solution (1 mg/mL):

4 µL of the 250 mg/mL FB28 stock solution was diluted in 996 µL of PBS. The solution was mixed by gentle vortexing and used fresh.

##### NucGreen^™^ Dead 488 ReadyProbes^™^ Reagent Working Solution:

The working solution was prepared by adding 2 drops of NucGreen^™^ Dead 488 ReadyProbes^™^ Reagent to 1 mL of PBS, pH 7.4. The solution was vortexed gently and used fresh.

##### Thioflavin T (ThT) Stock Solution (0.425 mg/mL):

A 50 mM Tris-HCl buffer (pH 8.0) was used for all ThT-related steps and was prepared by diluting a 0.5 M Tris-HCl stock solution 1:10 in distilled water. A stock solution (equivalent to 1 mM) was prepared by dissolving 4.25 mg of Thioflavin T (tech. grade, 75% purity) in 10 mL of the 50 mM Tris-HCl buffer. The solution was passed through a 0.2 µm syringe filter to remove any aggregates and stored in aliquots at −20°C, protected from light.

##### ThT Working Solution (10.6 µg/mL):

The working solution (equivalent to 25 µM) was prepared fresh by diluting 25 µL of the 1 mM stock solution in 975 µL of 50 mM Tris-HCl buffer.

#### Sequential Fluorescent Staining of the Biofilm

All staining and wash steps were performed at room temperature (approx. 20–25°C) and protected from light. Volumes of 200 µL per well were used for staining and washing in ibidi µ-Slide 8 Well high plates, unless otherwise indicated. Imaging commenced approximately 15 minutes after completion of the final staining step.

#### Sequential Multi-component Staining

##### NucGreen^™^ Dead 488 Staining (Nucleic Acids):

The PBS overlying fixed biofilms was removed. Biofilms were washed once with PBS. After aspirating the wash, NucGreen^™^ Dead 488 working solution was added to each well and incubated for 10 minutes. The staining solution was removed, and wells were washed 3 times with PBS.

##### Fluorescent Brightener 28 Staining (β-Polysaccharides):

Following the final PBS wash from NucGreen staining, PBS was removed. FB28 working solution (1 mg/mL) was added to each well and incubated for 10 minutes. The staining solution was removed, and wells were washed 3 times with DPBS.

##### Concanavalin A, Alexa Fluor^™^ 647 Staining (α-Polysaccharides):

Following the final DPBS wash from FB28 staining, DPBS was removed. After aspirating the final DPBS wash, ConA-647 working solution (200 µg/mL) was added to each well and incubated for 30 minutes. The staining solution was removed, and wells were washed 3 times with DPBS.

##### Sample Preparation for Imaging:

After the final wash, 200 µL of fresh DPBS was left in each well to keep the samples hydrated during microscopy.

#### Thioflavin T Staining for Functional Amyloids

For amyloid staining, fixed biofilms were stained separately. The PBS overlying the fixed biofilms was removed, and the wells were washed 3 times with 50 mM Tris-HCl buffer. After aspirating the wash, 200 µL of the 25 µM ThT working solution was added to each well and incubated for 30 minutes. The staining solution was subsequently removed, and the wells were washed three times with 50 mM Tris-HCl buffer to reduce background fluorescence. After the final wash, 200 µL of fresh 50 mM Tris-HCl buffer was left in each well to keep the samples hydrated during microscopy.

#### Minimum inhibitory concentration determination

Isoniazid, rifampicin, and (−)-epigallocatechin gallate (EGCG) were solubilized in dimethyl sulfoxide (DMSO). The compounds were serially diluted and 2 µl was spotted onto each well on a 96-well flat bottom plate. 2 µl DMSO was spotted onto solvent-control wells. Mid-log *Mtb* cultures were diluted to OD_600_ 0.005, and 200 µl culture was dispensed into each well. EGCG develops coloration over time, hence bacteria-free controls containing EGCG and media were included for background normalization. Light absorbance at 600 nm was measured in the spectrophotometer after 10 days incubation in a humidified incubator shaking at 100 rpm.

#### Bacterial viability assay in antibiotic challenge

*Mtb* biofilms in 24-well plates were prepared as described before, with the exception that the cell density was adjusted to OD_600_ 0.5 and 0.5 ml of culture was dispensed into each well. After 72 hours incubation, the biofilm media containing DTT was removed and the biofilms were washed once with sterile DPBS. Fresh biofilm medium (without DTT) containing various antibiotic or solvent (DMSO) was added to respective wells. For suspension cultures, mid-log *Mtb* cultures were centrifuged at 3,500 ×g for 10 minutes. The cell pellet was then washed once with the fresh medium and adjusted to cell density of OD_600_ 0.5. 5 ml of culture was dispensed into square 30 ml PETG bottles. DMSO, isoniazid, or rifampicin was added into each bottle. The final concentration of DMSO in the assay media was limited to 1% to prevent solvent-related toxicity effects. The biofilms were incubated in a humidified standing incubator, while the suspension cultures were kept shaking at 100 rpm. Bacterial viability was determined after 3, 6, and 9 days of incubation. 3 wells of biofilms from each condition were sacrificed at every timepoint: the biofilm was thoroughly scraped from the bottom of the well using a wide-bore pipette tip, and the entire contents transferred into a 1.5 ml snap cap tube. The tubes were centrifuged at 3,500 ×g for 10 minutes, and the supernatant was removed. 0.25 ml of PBS was added to each tube to resuspend the biofilm pellet, and briefly sonicated (10 seconds) in a water bath sonicator. 250 µl of suspension culture was sampled from each bottle and placed in 1.5 ml snap cap tubes. The tubes were centrifuged, resuspended and sonicated in the same workflow. The extracts were then serially diluted and plated on 7H10 agar plates with all necessary supplements. Bacterial viability was determined by enumerating the colony forming units after 21 days and accounting for the corresponding dilution factor.

### MICROSCOPY AND IMAGE ACQUISITION

#### Transmission Electron Microscopy imaging

Biofilm samples were fixed by immersion in 2.5% Glutaraldehyde in 0.1M cacodylate buffer (pH 7.4).

Samples were washed 3 times for 10 min in 0.1 M cacodylate buffer (pH 7.4), followed by post-fixation for 1 h on ice in reduced osmium solution prepared from potassium ferrocyanide, 0.1 M cacodylate buffer, and 2% osmium tetroxide. After washing in distilled water, samples were incubated for 40 min in freshly prepared thiocarbohydrazide solution, washed again, and post-fixed in 2% osmium tetroxide for 90 min at room temperature. Samples were then washed and stained overnight in 1% aqueous uranyl acetate at 4 °C. On the following day, samples were washed in distilled water, including three 10-min washes in prewarmed water at 50 °C, and incubated for 2 h at 50 °C in lead aspartate solution adjusted to pH 5.0. After washing, samples were dehydrated through a graded ethanol series (70%, 80%, 96%, and 100%; 20 min each), followed by two additional 15-min washes in absolute ethanol and two 15-min washes in propylene oxide. Samples were infiltrated overnight in a 1:1 mixture of Epon/Araldite and propylene oxide, transferred to 100% Epon/Araldite for 50 min, flat-embedded in fresh Epon/Araldite in Aclar foil, and polymerized at 60 °C for 28 h. Thin sections (70nm) were imaged in a Talos 120 transmission electron microscope at 120 kV acceleration voltage equipped with a bottom mounted Ceta camera using the Maps software for automatic image acquisition (Thermo Fischer Scientific, Eindhoven, The Netherlands).

#### Fluorescence imaging

Confocal microscopy was performed using a Leica TCS SP8 FALCON point-scanning confocal microscope system, built on a Leica DMI8 inverted microscope stand (Leica Microsystems). The system was equipped with a 405 nm solid-state laser, a pulsed White Light Laser (WLL; 470–670 nm tunable range), and Hybrid Detectors (HyD). Image acquisition was controlled using Leica Application Suite X (LAS X) software.

Imaging was conducted using a 63x immersion microscope objective (NA=1.3). Images were acquired with a resolution of 2048 × 2048 pixels (184.52 × 184.52 µm field of view; 90.14 × 90.14 nm pixel size) and 3x line averaging, using a scan speed of 600 Hz and a pinhole setting of 0.85 AU. Three-dimensional (3D) reconstructions were generated from Z-stacks acquired with a 1 µm step size through the depth of the biofilm.

For multi-component imaging, two separate acquisitions were performed in frame sequential mode to prevent spectral bleed-through. The first acquisition captured the polysaccharide signals, and the second captured the nucleic acid signal from the same field of view. The settings were as follows:

- **Calcofluor White (FB28):** Excitation: 405 nm. Emission detection: 430–500 nm.
- **Concanavalin A, Alexa Fluor**^**™**^ **647 (ConA-647):** Excitation: 653 nm (from WLL). Emission detection: 658–775 nm.
- **NucGreen**^**™**^ **Dead 488 (SYTOX**^**™**^ **Green):** Excitation: 504 nm (from WLL). Emission detection: 510–630 nm.

For separate amyloid detection experiments, the following settings were used:

- **Thioflavin T (ThT), bound:** Excitation: 405 nm. Emission detection: 480–530 nm.
- **Thioflavin T (ThT), free control:** Excitation: 405 nm. Emission detection: 415–460 nm.

Laser power for each channel was adjusted as needed to avoid detector saturation. Typical laser power settings were: **FB28 at 8%**, **NucGreen at 20%**, **ConA-647 at 1.7%**, and **ThT at 8%**. HyD detector gain was generally set to 80%. All imaging was performed in a dark room, commencing approximately 15 minutes after the final staining wash.

Confocal microscopy of PFA-inactivated, BSL-3 *Mtb* biofilms was performed on an inverted Leica SP8 point laser scanning confocal microscope using an HC PL APO CS2 63x/1.4 oil immersion objective and the LasX microscope-control software. Images were acquired with 405 nm laser excitation. Laser power and detector gain was generally adjusted to avoid signal saturation. Bound and free ThT detection settings were setup as mentioned previously.

#### QCL based Mid-Infrared (Mid-IR) Reflection Microspectroscopy

Biofilms of *Mtb* strains (AE1201, AE1601, AE1219, AE1611), cultured and fixed as previously described, were prepared for mid-IR imaging. Briefly, after fixation in 4% paraformaldehyde and rinsing with sterile distilled water, biofilm-coated mid-IR-compatible MirrIR low-e microscope slides (Kevley Technologies) were aseptically air-dried and stored at room temperature until analysis^90^.

Hyperspectral mid-IR imaging was performed using a Quantum Cascade Laser (QCL)-based mid-IR microscope (Spero^®^ QT 340, DRS Daylight Solutions) operating in reflection mode. The system is equipped with a tunable QCL source covering the fingerprint region from approximately 1800 cm^−¹^ to 950 cm^−¹^ and a 480×480 focal plane array (FPA) detector, enabling a spatial resolution of 12 µm at a wavelength of 5.5 µm as well as rapid data acquisition (~1 minute per hyperspectral cube). Reflection mode was chosen for its suitability with fixed biofilm samples on the reflective Kevley slides.

Hyperspectral data cubes were acquired using the SperoChemVision^™^ software (Daylight Solutions). Data processing was conducted using a custom Python-based pipeline. To mitigate baseline variability arising from sample thickness heterogeneity and optical scattering effects, spectra at each pixel were z-score normalized: each spectrum's mean intensity was subtracted, and the result was divided by its standard deviation across the 950–1800 cm^−¹^ range. No further baseline correction algorithms were applied post-z-score normalization.

### DATA PROCESSING AND ANALYSIS

Color overlay images were generated using Fiji (ImageJ). For merged images presented in Figure 2d, h, l and p, a threshold was applied to the lower intensity range of the green channel (nucleic acid signal) to enhance the visualization of brighter bacilli against any diffuse background signal.

#### Generation of Thresholded Specific Wavenumber Abundance Maps (TSWAMs)

To visualize the spatial distribution of specific biomolecular signatures, TSWAMs were generated from the z-score normalized hyperspectral data. For a chosen wavenumber:

1. The z-score normalized intensity values at that specific wavenumber across all pixels in the 2D image were linearly scaled to a range of 0 to 1.
2. A binary mask was created by applying an intensity threshold, typically selecting pixels with normalized intensity values >0.55 (representing the top 45% abundance), unless otherwise stated.
3. The intensity values (from step 1) within this binary mask were then re-normalized to a 0–1 range (e.g., original 0.55–1 range scaled to 0–1) to enhance the dynamic range for visualization in the final maps. These TSWAMs, representing regions of higher relative abundance for selected wavenumbers, were used for qualitative assessment of biochemical distribution and compartmentalization. The approximate field of view (FOV) for these images was 1.4 × 1.4 mm.

#### Principal Component Analysis (PCA) of Mid-IR Hyperspectral Data

Prior to clustering, Principal Component Analysis (PCA) was applied to the z-score normalized mid-IR hyperspectral data cubes (950–1800 cm^−¹^, one spectrum per pixel) for each *Mtb* strain to reduce dimensionality and noise. The data were then scaled using StandardScaler from scikit-learn. PCA was performed using sklearn.decomposition. The number of principal components (PCs) retained for subsequent clustering was determined by the Kaiser criterion, i.e., selecting PCs with eigenvalues greater than 1.

#### K-means Clustering and Cluster Evaluation

K-means clustering was performed on the retained PCs for each *Mtb* strain dataset using the KMeans function from sklearn.cluster. The algorithm was run with 10 different initializations (n_init=10) and a fixed random state (random_state=42) for reproducibility, selecting the run with the lowest inertia. To determine the optimal number of clusters (k) for each strain, a range of k values from 2 to 15 was evaluated using several common clustering metrics:

1. **Inertia (Elbow Method):** The sum of squared distances of samples to their closest cluster center was plotted against k to identify an “elbow” point, suggesting a suitable k.
2. **Davies-Bouldin Score:** This metric measures the average similarity ratio of each cluster with its most similar cluster. Lower values indicate better clustering, with a minimum score of 0.
3. **Calinski-Harabasz Index (Variance Ratio Criterion):** This index is the ratio of the sum of between-cluster dispersion to within-cluster dispersion. Higher values generally indicate better-defined clusters.

The final number of clusters for each strain (AE1201: k=4; AE1219: k=3; AE1601: k=3; AE1611: k=3) was chosen by seeking a consensus among these metrics.

#### Generation of Cluster Assignment Maps and Average Spectra

Cluster assignment maps were generated by assigning each pixel in the original spatial dimensions to the cluster determined by K-means analysis of its corresponding PCA-reduced spectrum. Average cluster spectra were then calculated for each identified cluster by averaging the original z-score normalized mid-IR spectra (950–1800 cm^−¹^) from all pixels assigned to that cluster.

### SOFTWARE AND CODE AVAILABILITY

Data processing and analysis were performed using Python. Key libraries included scikit-learn for PCA and K-means clustering, NumPy for numerical operations, and Matplotlib for plotting. Custom Python scripts used for PCA, clustering metric calculation, and parts of the data visualization will be made available upon publication at e.g. a GitHub repository link or other public archives.

## Extended Data
